# Rehabilitation Involving Tailored Subsymptomatic Aerobic Exercise in Adult Patients With Persistent Postconcussion Symptoms (REPCon Project): Protocol for an Assessor-Blinded, Parallel-Group, Randomized Controlled Trial

**DOI:** 10.2196/93795

**Published:** 2026-06-04

**Authors:** Bodil Wiberg Larsson, Mette Kreutzfeldt Zebis, Jakob Vismann, Frederik Friis Ørner, Edis Devin Tireli, Kenneth Damkjær Clausen, Isabel Fenger, Daniel Alstrup Shacham, Derya Tireli, Bjarki Thor Haraldsson, Stig Præstekjær Cramer, Ulrich Lindberg, Mark Bitsch Vestergaard, Hilde Sylliaas, Faisal Mohammad Amin, Henrik Bo Wiberg Larsson

**Affiliations:** 1 Department of Midwifery, Physiotherapy, Occupational Therapy and Psychomotor Therapy, Faculty of Health, University College Copenhagen, Sigurdsgade 26, Copenhagen, Capital Region, 2200 N, Denmark, 45 40318993; 2 Institute of Sports Medicine Copenhagen, Department of Orthopaedic Surgery, Copenhagen University Hospital – Bispebjerg and Frederiksberg, Copenhagen, Denmark; 3 Department of Physical and Occupational Therapy, Hvidovre Hospital, Copenhagen, Denmark; 4 Functional Imaging Unit, Department of Clinical Physiology and Nuclear Medicine, Copenhagen University Hospital – Rigshospitalet, Glostrup, Denmark; 5 Department of Clinical Medicine, Faculty of Health and Medical Science, University of Copenhagen, Copenhagen, Denmark; 6 Center of Rehabilitation and Emergency Care, Copenhagen, Denmark; 7 Fysisk Form Amagerbro, Copenhagen, Denmark; 8 Center of Rehabilitation, Vanløse, Copenhagen Municipality, Copenhagen, Denmark; 9 Department of Radiology, Scanning and Nuclear Medicine, Amager and Hvidovre Hospital, Copenhagen University Hospital, Copenhagen, Denmark; 10 Department of Public Health Science, Norwegian University of Life Sciences, Ås, Akershus, Norway; 11 Department of Neurology, Copenhagen University Hospital - Amager and Hvidovre Hospital, Hvidovre, Denmark

**Keywords:** persistent postconcussion symptoms, graded aerobic exercise, randomized controlled trial, functional magnetic resonance imaging, patient perspective, 360-degree evaluation

## Abstract

**Background:**

Persistent postconcussion symptoms (PPCSs) represent a complex phenomenon following mild traumatic brain injury (mTBI), and this phenomenon is characterized by a diverse range of symptoms that greatly impact daily functioning, including the ability to engage in physical, cognitive, and social activities. Effective rehabilitation strategies and a nuanced understanding of brain function and patient perception remain insufficiently explored in adults with PPCSs.

**Objective:**

This study aims to assess the physiological and neurofunctional effects of targeted physical exercise carried out as a graded subsymptomatic aerobic exercise program. Furthermore, the project seeks to alleviate the complex symptomatology and perception of PPCSs through the planned intervention. In addition, the study aims to obtain feedback from patients and researchers involved in this interprofessional project and to conduct a comprehensive 360-degree evaluation combining subjective and objective data in order to obtain novel insights into the symptom complexity of PPCSs, ultimately paving the way for future strategic initiatives in mTBI treatment.

**Methods:**

This randomized controlled trial enrolled 70 patients with PPCSs. The patients were randomized in a 1:1 ratio to either a control group or a training group. Patients in the control group maintained their usual activities and care. Patients in the training group underwent a 12-week tailored aerobic exercise program designed to stay below symptom-provocation thresholds, using the Buffalo Concussion Bike Test (BCBT) as the assessment instrument. The study consisted of qualitative patient interviews, physical tests, exercise evaluations, neurofunctional and structural magnetic resonance imaging (MRI) scans, and process evaluations. MRI assessed neurophysiological changes, including blood-brain barrier integrity and cerebral metabolism. The primary outcome is improvement in physical performance on the BCBT. The secondary outcomes are symptom severity, neurophysiological function, and quality of life. The study will incorporate a multimodal approach, combining subjective patient reports with objective clinical and neuroimaging data, enabling the interdisciplinary research group to perform comprehensive evaluations of the complexity of PPCSs.

**Results:**

Study funding was awarded in December 2022. Enrollment and randomization of 70 patients were completed in March 2025. The 12-week postintervention assessments concluded in August 2025. Data analysis is ongoing and expected to conclude in 2026. The results will be published in separate manuscripts in 2027.

**Conclusions:**

This study is innovative in its approach to patients with PPCSs, focusing on the effect of an individualized exercise regimen and exploring the neurofunctional underpinnings of symptom improvements as well as the patient perceptions of dealing with PPCSs. The findings are anticipated to contribute greatly to the field of PPCS management, potentially transforming current rehabilitation practices.

## Introduction

### Background

Persistent postconcussion symptoms (PPCSs) may develop in some individuals after mild traumatic brain injury (mTBI) [1,2], and mTBI can be used interchangeably with “concussion,” as defined by the American Congress of Rehabilitation Medicine in 2023 [3,4]. The symptoms represent a complex phenomenon causing a broad spectrum of issues, including musculoskeletal and balance disturbances, cognitive impairments, visual and auditory dysfunctions, fatigue, and headache-related problems [5]. Adults with PPCSs frequently demonstrate increased sensitivity to physical, cognitive, and perceptual stimuli, which can substantially impair their capacity to engage in physical activity, participate in social interactions, sustain full-time employment, and pursue or complete higher education [6].

In Denmark, approximately 30,000 to 40,000 adults experience traumatic brain injury (TBI) annually, with more than 80% of cases being classified as mTBI [6,7]. However, this number is likely underestimated owing to underreporting and the fact that only hospital contacts are included in the epidemiological statistics. Within the subset of approximately 25,000 individuals with mTBI, 40% experience symptoms persisting beyond 4 weeks, and up to 15% continue to experience symptoms up to a year or longer after the initial concussion [6]. A nationwide cohort study identified 19,732 adults aged 18‐60 years with hospital-diagnosed mTBI over 5 years from 2003 to 2007, and found that 43% of the individuals were not engaged in full-time employment 5 years after mTBI [6].

TBI involves damage to brain tissue, hemorrhage, and neurometabolic disorders [8]. In contrast, mTBI is characterized by normal imaging findings, despite the presence of complex and disabling symptoms [9]. Symptom aggravation in patients with PPCSs is unpredictable in terms of timing and severity, often impeding the ability to lead a normal everyday life. In some cases, it may even be impractical or futile to pursue basic activities. There appears to be a threshold beyond which postconcussive symptoms worsen, potentially due to intolerance to various stimuli and activities. Symptom aggravation may be caused by microtraumas in the brainstem and corpus callosum [10,11]. Symptoms of exercise intolerance are often part of the clinical presentation, and symptom-limited exercise cessation serves as an indicator of this intolerance, reflecting the underlying pathophysiology of PPCSs. Concussions are thought to disrupt autonomic nervous system regulation of cerebral blood flow (CBF), producing abnormal cardiovascular and cerebrovascular responses to increasing metabolic demands that are not detectable at rest [12,13]. A graded exercise test is capable of provoking this dysregulation in a controlled and systematic way [5,12]. By adopting the same operational definitions as Leddy et al [13] and Haider et al [14], a direct comparison with prior work using both the Buffalo Concussion Treadmill Test and the Buffalo Concussion Bike Test (BCBT) is possible.

National clinical guidelines for the nonpharmacological treatment of persistent symptoms following mTBI recommend graded aerobic physical exercise as an important part of rehabilitation; however, these guidelines also underscore the lack of evidence in the existing literature [2]. This highlights the need for further clinical controlled studies to better understand this field, including the pathophysiology of brain metabolism and blood-brain barrier (BBB) permeability [15] in association with functional ability, as well as research into patient perceptions of PPCSs.

The body of literature on PPCSs consists mainly of a limited number of randomized controlled trials (RCTs), with a disproportionate focus on the recovery of elite youth athletes following sport-related concussions [1,16-19]. This research bias significantly limits a comprehensive understanding of the manifestation of PPCSs in the general population. Furthermore, literature often overlooks the daily challenges and symptoms reported by patients in their routine lives. The evaluation of therapeutic interventions frequently lacks the integration of patient perspectives. Thus, an interdisciplinary approach that combines patient-experienced knowledge with individualized therapeutic exercise programs may provide deeper insights into postconcussion cerebral functioning. This approach has the potential to substantially advance the understanding of the field. This could lead to more informed development, implementation, and longitudinal evaluation of intervention methodologies, as well as highlight patient perceptions throughout the complex trauma of PPCSs.

The purpose of this interprofessional RCT is to investigate the effect of a 12-week graded aerobic physical exercise program combined with usual activities and/or care for the treatment of adult patients with PPCSs. Our approach provides an opportunity to triangulate subjective and objective patient data with regard to novel combinations of patient perception, physiology, and neurobiology. The overall study perspective is to establish a tailored, evidence-based active treatment for PPCSs that benefits patients and supports clinical decision-making, and to explain the pathophysiology of PPCSs.

### Objectives

This RCT evaluates the effect of an individualized graded aerobic exercise intervention in adults with PPCSs from the general population. Participants undergo an intensive battery of physical and magnetic resonance imaging (MRI) examinations, alongside personal interviews and process evaluations, throughout the intervention period. The project is divided into the following 4 subprojects:

- Subproject 1: This subproject aims to understand the challenges faced by patients with PPCSs, particularly their potential intolerance to physical activity and exercise, through qualitative in-depth interviews. It is hypothesized that the interviews will reveal significant obstacles to living a normal life, with a focus on intolerance to activity and exercise.
- Subproject 2: This subproject aims to evaluate the efficacy of a 12-week, heart rate–controlled aerobic exercise program for patients with PPCSs. It is hypothesized that the specialized exercise program will increase the physical capacity of patients, raising the threshold of symptom provocation compared with a group of controls.
- Subproject 3: This subproject aims to quantify the neurofunctional aspects of PPCSs using MRI for measuring changes in brain structure and function. It is hypothesized that patients with PPCSs will exhibit increased BBB permeability, disturbances in brain metabolism, and abnormalities in brain structure and physiology compared with a group of healthy age-matched controls. The exercise intervention is expected to normalize the physiological aspects of these MRI metrics in the training group (TG).
- Subproject 4: This subproject aims to obtain qualitative feedback from patients and researchers on various aspects of this interprofessional scientific project, utilizing a strengths, weaknesses, opportunities, and threats (SWOT) framework. Finally, we will conduct a comprehensive 360-degree evaluation that combines subjective and objective data to attain a novel understanding of the complexity of PPCSs.

## Methods

### Trial Design

This study protocol describes the design of the Rehabilitation in Postconcussion Symptoms (REPCon) project, an assessor-blinded, parallel-group, randomized controlled superiority trial. Adult patients with PPCSs showing symptoms for more than 4 weeks after the initial mTBI were randomized in a 1:1 ratio to either a control group (CG) or a TG. Participants in both groups continued their usual daily activities and received usual care as delivered in routine clinical practice. In accordance with the pragmatic design of the trial, usual care is not standardized and reflects current real-world management. Usual care may include follow-up by a general practitioner or medical specialist, symptom monitoring, pharmacological treatment (eg, for pain or sleep disturbances), advice regarding activity modification and return to activity, and referral to allied health services such as physiotherapy or psychology, as clinically indicated. Participants allocated to the TG additionally received a supervised graded physical exercise program, as described below. Concomitant care and co-interventions were discouraged in both groups, and any changes were reported to the study group. The only protocol-mandated difference between groups was the addition of a supervised exercise intervention in the TG. The study includes the following four subprojects: (1) qualitative evaluation, (2) physiological evaluation, (3) neurofunctional evaluation, and (4) comprehensive evaluation. The primary outcome is improvement in exercise tolerance measured as time to symptom exacerbation during the graded aerobic exercise testing protocol 12 weeks after baseline [14]. The secondary outcomes include differences in symptom complexity, dynamic gait, fatigue, headache, and the findings of a range of other neurophysiological tests and functional abilities between the TG and CG at 12 weeks after baseline, as well as the normalization of quantitative MRI measures in the TG and the between-group differences in these measures. The tertiary outcome is the triangulation of data from the subprojects to provide a nuanced and integrated understanding of biology, physiology, and patient perceptions of PPCSs, thereby potentially improving clinical recommendations. The trial has been registered in the ClinicalTrials.gov repository (NCT05785000). The findings will be reported in accordance with the SPIRIT (Standard Protocol Items: Recommendations for Interventional Trials) guidelines [20,21]. The SPIRIT checklist is provided in Checklist 1.

### Setting

The REPCon project involved 2 settings. The first setting was the House of Practice & Innovation at the University College Copenhagen, Denmark, where data collection for qualitative interviews, physical tests, the exercise intervention of the TG, and process evaluations took place. The qualitative interviews and process evaluations were performed by the project leader (BWL) and the Norwegian research partner (HS). The physical tests were conducted by physiotherapists (JV, FFØ, KDC, IF, and DAS) and guided by the project leader (BWL). All exercise sessions were led by personal trainers with academic backgrounds in physiology (BTH) and physiotherapy or by physiotherapy students and were guided by the project leader (BWL).

The second setting was Rigshospitalet, Copenhagen, Denmark, where the imaging procedure (magnetic resonance [MR] scans) was conducted under the supervision of the principal investigator (HBWL), physicians (SPC and DT), a physicist (ET), engineers (MBV and UL), and radiographers.

### Eligibility Criteria

The eligibility criteria for trial participation are presented in Textbox 1.

Textbox 1. **Inclusion and exclusion criteria of the study.**

**Inclusion criteria**

- Age 18-70 years
- At least one mild traumatic brain injury
- Medical documentation according to the 10th revision of the World Health Organization’s International Classification of Diseases
- Postconcussion symptoms for more than 4 weeks after trauma
- Proficient in the Danish language

**Exclusion criteria**

- Moderate/severe traumatic brain injury
- Nonavailability for 12 consecutive weeks
- Contraindication for magnetic resonance imaging, including contrast agents
- Medical conditions that can obscure postconcussion symptoms
- Cardiovascular diseases that prohibit the planned physical exercise activity
- Professional or elite athlete

### Recruitment and Screening

Patients were recruited through a network of multidisciplinary professionals with an established referral flow of adults with PPCSs, including general practitioners, physiotherapy clinics, municipal rehabilitation centers, and the Danish Patient Association for Concussion. Additional recruitment occurred via internal bulletins at the University of Copenhagen and University College Copenhagen, as well as through online postings on the recruitment platform TrialTree. Interested individuals received comprehensive written information about the study and were invited to participate in an initial telephone screening interview completed by the project leader (BWL) and the physiotherapists (JV and KDC). During this interview, a structured questionnaire was administered to assess eligibility based on the predefined inclusion and exclusion criteria. Eligible candidates were subsequently invited to an in-person clinical screening examination at University College Copenhagen, conducted by a medical doctor (HBWL, SPC, or DT) and a physiotherapist (BWL or JV). This session included verification of medical documentation of mTBI, a clinical neurological examination, completion of screening questionnaires, and provision of detailed verbal and written information about the study, including information about how to contact the medical doctor and project leader throughout the project. All patients provided written informed consent prior to enrollment into the trial.

### Randomization and Blinding

Randomization to either the TG or the CG occurred after completion of the clinical screening examination and followed a 1:1 allocation ratio. Because PPCSs are more prevalent among women than men, an equal sex distribution was not anticipated; however, the allocation ensured an equal number of patients with PPCSs in each group. The randomization sequence was generated by MKZ, who was not involved in any other aspect of the trial, including patient recruitment, assessment, intervention delivery, data management, or analysis, thereby maintaining independence and minimizing the risk of allocation bias. The sequence was produced a priori using a computer-generated randomization list created through an online tool provided by Sealed Envelope. Allocation was implemented according to this pregenerated list, ensuring that those responsible for enrolling patients and assigning them to groups had no access to the randomization sequence prior to assignment, which is in line with SPIRIT recommendations for allocation concealment.

For all baseline assessments in subproject 2 (physiological evaluation), the tester was blinded to group allocation. The tester was also blinded to group allocation for all 12-week follow-up assessments. In addition, different testers conducted the baseline and 12-week follow-up assessments. Furthermore, the personal trainers supervising the exercise sessions were different from all the testers.

In subproject 3 (neurofunctional evaluation), which involved collecting and analyzing MRI data, a blinding protocol was strictly followed. The project staff handling and analyzing the MRI data were unaware of the participants’ group allocations. Similarly, the project leaders and statisticians were blinded to group allocations in the dataset used for the physiological and neurobiological analyses.

### Physical Tests

All participants completed a multimodal battery of physiological tests (Table 1) at baseline and the 12-week follow-up. Since all participants had completed a subjective symptom profile assessment and a basic neurological test at screening, the purpose of the physical test was to cover relevant physical, oculomotor, balance, autonomic, and cervical aspects of PPCSs through objective measures prior to the validated BCBT [14]. The BCBT determined the individual HR threshold at which concussion-specific symptom exacerbation occurred and outlined the structure for the 12-week training program in the TG.

**Table 1.** Overview of data collection, prescreening, clinical screening, and physiological tests in the study.

Variable	Week 0	Week 4	Week 8	Week 12
Prescreening phone interview				
Trauma description	✓	—^a^	—	—
Symptoms reported at prescreening	✓	—	—	—
Job situation	✓	—	—	—
Clinical screening				
Neurological examination	✓	—	—	—
Symptoms described at screening	✓	—	—	—
Irritability	✓	—	—	—
Cervical spine subjective description	✓	—	—	—
Headache description	✓	—	—	—
Vestibular and oculomotor	✓	—	—	—
Symptoms beyond physical issues	✓	—	—	—
Physical activity	✓	—	—	—
Physiological tests				
Posture	✓	—	—	✓
VAS^b^ (beginning of the test)	✓	—	—	✓
Cervical range of motion	✓	—	—	✓
Cervical safety test	✓	—	—	✓
Tendon reflexes	✓	—	—	✓
Dynamic Gait Index	✓	—	—	✓
Smooth pursuit for nystagmus	✓	—	—	✓
Repetitive saccades	✓	—	—	✓
Fatigue Severity Scale	✓	—	—	✓
VAS inverse	✓	—	—	✓
RPQ^c^	✓	✓	✓	✓
Orthostatic blood pressure	✓	—	—	✓
VAS (before the BCBT^d^)	✓	✓	✓	✓
Extended description of VAS	✓	—	—	✓
BCBT	✓	✓	✓	✓
Time cycled	✓	✓	✓	✓
Watt (end of the test)	✓	✓	✓	✓
Pulse rate (end of the test)	✓	✓	✓	✓
Rate of perceived exertion (end of the test)	✓	✓	✓	✓
VAS (end of the BCBT)	✓	✓	✓	✓

a Not applicable.

b VAS: Visual Analog Scale.

c RPQ: Rivermead Post-Concussion Symptoms Questionnaire.

d BCBT: Buffalo Concussion Bike Test.

### Physical Intervention: Graded Aerobic Exercise

Participants with PPCSs randomized to the TG participated in an individualized 12-week graded aerobic exercise program performed on an ergometer bicycle. The intervention consisted of 2 supervised sessions per week (24 sessions in total) and was delivered in addition to participants’ usual care and regular activities. In line with procedures applied in the CG, participants were instructed to maintain all ongoing treatments and regular activities throughout the 12-week intervention period and were not permitted to initiate any new treatments or activities during this time. This requirement was clearly communicated at screening visits and prior to the blinded random allocation as a condition for study participation. Each exercise session lasted 45 minutes (20 minutes for the specific exercise session on the bike and 25 minutes for filling out questionnaires and practicalities), with at least 1 day of rest between sessions. All sessions were conducted at the House of Practice & Innovation, University College Copenhagen, Denmark, and were supervised by exercise physiologists, physiotherapists, and physiotherapy students, all of whom had received standardized training in the delivery of the graded aerobic exercise protocol. At the beginning of each session, patients reported symptoms experienced since the previous session, rated their current symptom severity using a Visual Analog Scale (VAS) [14], and completed the Rivermead Post-Concussion Symptoms Questionnaire (RPQ) [22]. Sessions commenced with a 2-minute warm-up at light resistance (60 revolutions per minute [rpm]), corresponding to 50% of the heart rate at symptom threshold (HRt), as determined by the BCBT [14] conducted at baseline. The BCBT was repeated at weeks 4 and 8 to reassess the HRt and allow individual adjustment of exercise intensity. The main training phase consisted of ergometer cycling at 60 rpm for 20 minutes, with intensity set at 70%‐80% of the HRt. HR, Borg Rating of Perceived Exertion (RPE) [14,23], VAS symptom rating, pulse oximetry results, and watt output were recorded at 0, 7, 14, and 20 minutes during each session. Sessions concluded with a 2-minute cool-down at 50% of the HRt, followed by a brief evaluation of the perceived energy level and symptom response. Exercise intensity and physiological responses were monitored continuously to assess symptom provocation. Training was individually adjusted based on subjective symptom reporting (VAS) and perceived exertion (Borg RPE). As part of a standardized protocol, cervical posture, shoulder girdle tension, and breathing patterns were monitored and corrected to ensure an appropriate cycling environment.

Exercise was terminated immediately if any of the following criteria were met: (1) exacerbation of PPCSs, defined as a ≥3-point increase on the VAS; (2) a HR exceeding 90% of the HRt; or (3) a desire or need to discontinue the session.

### Adherence

Adherence to the study protocol was monitored throughout the 12-week intervention period. Participants in both the TG and CG reported their perceived well-being and exercise activity twice weekly using mail or SMS text messages. In these messages, participants specified the type and duration of their rehabilitation and physical activities. Automated reminder messages were sent in cases of missing responses.

### Qualitative Data Collection and Analysis

Subprojects 1 and 4 were epistemologically informed by phenomenological [24] and constructivist [25,26] perspectives, aiming to capture participants’ lived experiences of PPCSs while highlighting the process of participating in a clinical study. During the 2-year phase of the overarching data collection, qualitative interviews were performed with 6 participants, and data were generated through open-ended interviews that encouraged rich first-person descriptions and paved the way for a second round of qualitative interviews [25]. Following the 2-year phase of data collection, all participants were invited for a second round of individual interviews. A total of 44 participants completed these interviews, and data were generated through a semistructured interview guide. Analytically, data will be examined using the Charmaz constructivist grounded theory methodology [25]. The analysis will proceed through iterative cycles of initial and focused coding, constant comparison, and memo writing. In vivo codes will be used where appropriate to retain participants’ own language, supporting the development of interpretive categories grounded in the empirical data and leading to the development of a conceptual model of living with PPCSs and participating in a clinical trial.

### MRI Protocol

The participants completed 2 MRI sessions (scan sessions A and B) at baseline and after the intervention period. An overview of MRI data collection is shown in Table 2. The MRI scans were performed at the Department of Clinical Physiology and Nuclear Medicine, Rigshospitalet, using a Philips 3T dStream Achieva MRI scanner with a 32-channel phased-array head coil.

**Table 2.** Overview of data collection and MRI^a^ measurements in the study.

Variable	Week 0	Week 4	Week 8	Week 12
Diffusion				
Mean diffusivity and anisotrophy	✓	—^b^	—	✓
Blood-brain barrier permeability	✓	—	—	✓
Cerebral blood flow	✓	—	—	✓
Capillary transit time heterogeneity	✓	—	—	✓
MR^c^ spectroscopy				
Brain lactate (normoxia)	✓	—	—	✓
Brain lactate (hypoxia)	✓	—	—	✓
MRI cerebral flow measurement by phase-velocity mapping and susceptometry				
Global mean cerebral blood flow (normoxia)	✓	—	—	✓
Global mean cerebral blood flow (hypoxia)	✓	—	—	✓
Global mean oxygen consumption (normoxia)	✓	—	—	✓
Global mean oxygen consumption (hypoxia)	✓	—	—	✓
MRI arterial spin labeling and visual stimulation				
Occipital cerebral blood flow (rest)	✓	—	—	✓
Occipital cerebral blood flow (visual stimulation)	✓	—	—	✓

a MRI: magnetic resonance imaging.

b Not applicable.

c MR: magnetic resonance.

Scan session A involved the acquisition of high-resolution structural brain images using a 3D T1-weighted turbo field echo sequence. The total brain volume, gray matter volume, white matter volume, and cortical thickness were estimated through the segmentation of the brain using standard software packages such as Freesurfer. A fluid-attenuated inversion recovery (FLAIR) sequence was used to acquire brain images for the assessment of the white matter lesion burden. Susceptibility-weighted images were obtained for the assessment of possible microbleeds or larger hematomas. Diffusion-weighted imaging was performed with b-values of 500, 1500, and 4000 s/mm^2^ in 6, 6, and 15 different diffusion encoding directions, respectively, allowing restricted spectrum analysis of brain tissue integrity. Finally, the BBB was measured with dynamic contrast-enhanced (DCE) T1-weighted MRI, using a standard dose of a conventional MRI contrast agent. BBB permeability was calculated using the Patlak method, where the slope of the Patlak plot (Ki) is an estimate of the unidirectional influx constant of the contrast agent as a marker of BBB permeability and can be interpreted as the permeability surface area product of the BBB with reference to the contrast agent. An in-depth description of the methods and calculations has been provided in previous publications [27,28].

Scan session B focused on a hypoxic stress test. CBF, cerebral metabolic rate of oxygen (CMRO2), and brain lactate concentration were obtained during normoxic conditions, and measurements were repeated during inhalation of hypoxic air containing 12%‐14% fractional oxygen. The hypoxic condition was created by connecting the participant through a 1-way valve and wide-bore tubing to a face mask covering both the nose and mouth and to an AltiTrainer system (SMTEC) that delivered hypoxic air. Throughout the scan, HR, blood pressure, arterial saturation (SaO2), inspired and expired oxygen partial pressures, and end-tidal carbon dioxide were continuously monitored using a Veris 8600 vital signs monitor (MEDRAD Inc). The hypoxic state lasted about 20 minutes. The initial 6‐7 minutes were used for ensuring a hypoxic steady state, and this was followed by repeated MRI measurements.

CBF was determined by measuring the blood flow through the feeding cerebral arteries using velocity-sensitive phase-contrast mapping MRI. Blood flow in the cerebral arteries was calculated by multiplying the mean blood velocity by the cross-sectional area of regions of interest defining each vessel. Total CBF was normalized to individual brain volume for each participant to yield CBF values in mL/100 g/min.

CMRO2 was calculated using the Fick principle as follows:


$$CMRO_{2}=Hgb\cdot CBF\cdot (SaO_{2}-SvO_{2})$$

The venous oxygen saturation (SvO2) of the blood leaving the brain in the sagittal sinus was measured using the susceptibility-based oximetry MRI technique. SaO2 was obtained by finger pulse oximetry and is known to be close to arterial oxygen saturation. An in-depth discussion of the sequences and postprocessing methods has been reported previously [29-33].

Cerebral lactate concentrations were measured using magnetic resonance spectroscopy (MRS) with a water-suppressed point-resolved spectroscopy pulse sequence. The MRS voxel was placed in the precuneus, and the sequence was optimized to measure lactate. Postprocessing and quantification were performed using LCModel (Version 6.3‐1F). The water peak measured in the spectrum was used to quantify the lactate concentration. The water concentration in the voxel was calculated based on the proportions of gray matter, white matter, and cerebrospinal fluid in the voxel identified by tissue segmentation of the anatomical images [33,34].

A 2D gradient-echo, dual-echo pseudo-continuous arterial spin labeling (pCASL) sequence with an echo-planar imaging readout was used. This sequence involved 2 echo times, 54 dynamics, and a total duration of 8 minutes for visual stimulation that consisted of a flickering black-and-white checkerboard presented to the participant inside the MR scanner. The flickering checkerboard was presented 4 times in 54-second blocks, and a 54-second period with a black screen was present before and after the session and in between each stimulation block. The flickering frequency was set to 8 Hz, which is known to result in the highest metabolic response [35]. CBF maps were obtained by subtracting blood-labeled and nonlabeled control images, and blood oxygenation level–dependent (BOLD)-weighted maps were calculated from the nonlabeled images acquired at the second long echo. The methodology has been described previously [35].

### Outcome Measures

#### Primary Outcome

The primary outcome measure is the change in exercise tolerance, assessed as the time (in minutes) to symptom exacerbation during the BCBT [14], following the 12-week graded aerobic exercise intervention compared with baseline. All BCBT assessments were conducted on the same Monark cycle ergometer (Monark Exercise AB) at both baseline and the 12-week follow-up. During each test, participants were instructed to maintain a pedaling cadence of 60 rpm while the workload increased incrementally every 2 minutes according to the standardized BCBT protocol. HR, VAS, and Borg RPE were recorded at the end of each 2-minute stage. The test continued until the participant reached voluntary exhaustion or experienced symptom exacerbation consistent with PPCSs, and this was followed by a 2-minute cooldown at the initial resistance level at a self-selected cadence.

#### Secondary Outcomes

Secondary outcomes have been assessed as between-group differences in change scores from baseline to postintervention (at 12 weeks), unless otherwise specified.

The officially approved Danish version of the *RPQ* [22] was used to assess changes in each patient’s discomfort specifically related to concussion at both baseline and 12 weeks. Furthermore, the questionnaire was used in the TG before each training session during all 12 weeks. It is a standardized questionnaire that evaluates patient perceptions of postconcussive symptoms experienced during the previous 24 hours. Scores range from 0 (not experienced) to 4 (experienced as a severe problem) for each of the 16 items in the questionnaire, with a total score of 64 points.

The *Fatigue Severity Scale* [36] was used to assess changes in each patient’s perceived fatigue both at baseline and after 12 weeks. It is a standardized questionnaire that evaluates fatigue severity and its impact on daily functioning. Scores range from 1 (strongly disagree) to 7 (strongly agree) for each of the 9 items in the questionnaire, with a total score of 63 points.

The *Dynamic Gait Index* [37] was used to assess changes in each patient’s dynamic and functional balance during gait at both baseline and 12 weeks. It is a standardized clinical tool to evaluate how an individual responds to walking challenges. Scores range from 3 (normal performance) to 0 (severe impairment) for each of the 8 items in the index, with a maximum score of 24 points.

*BCBT changes* [14] were assessed from baseline to week 4 and from baseline to week 8. The test followed the same protocol as described for the primary outcome and was completed for re-evaluation of the exercise level in the TG.

*Physical ability* was assessed twice weekly for 12 weeks using mail or SMS text message correspondence, in which patients electronically completed a researcher-developed questionnaire on self-reported physical ability, which was inspired by a previous study [38]. The questionnaire was rated on a scale from 0 to 10, with 10 representing the best physical ability.

*Overall physical symptoms* were assessed twice weekly for 12 weeks using mail or SMS text message correspondence, in which patients electronically completed a researcher-developed questionnaire on self-reported physical symptoms, which was inspired by a previous study [38]. The questionnaire was rated on a scale from 0 to 10, with 10 representing the worst physical symptoms.

*Cognitive engagement* was assessed twice weekly for 12 weeks using mail or SMS text message correspondence, in which patients electronically completed a researcher-developed questionnaire on self-reported cognitive engagement, which was inspired by a previous study [38]. The questionnaire was rated on a scale from 0 to 10, with 10 representing the best cognitive engagement.

*Workability* was assessed twice weekly for 12 weeks using mail or SMS text message correspondence, in which patients electronically completed a researcher-developed questionnaire on self-reported workability, which was inspired by a previous study [38]. The questionnaire was rated on a scale from 0 to 10, with 10 representing the best workability.

*Quality of daily living* was assessed twice weekly for 12 weeks using mail or SMS text message correspondence, in which patients electronically completed a researcher-developed questionnaire on self-reported quality of daily living, which was inspired by a previous study [38]. The questionnaire was rated on a scale from 0 to 10, with 10 representing the best quality of daily living.

*BBB permeability* was determined using DCE MRI. Tracer kinetic models calculated regional BBB permeability, blood supply, tissue perfusion, blood volume, and the distribution of capillary transit times [27,28,39,40]. Changes in BBB permeability were measured in mL/min/100 g of brain tissue in relation to healthy controls and patients with PPCSs and in relation to group (TG or CG) allocation.

*Brain oxygen consumption* change was assessed as described above in relation to group (TG or CG) allocation and the conditions of normoxia and hypoxia. Brain oxygen consumption was measured in µmol/min/100 g of brain tissue [29-33].

*Brain diffusion*, which indicates the change in the brain’s microstructural composition, was investigated using the restricted spectrum imaging diffusion method. Changes in diffusion metrics in relation to group (TG or CG) allocation were assessed with the diffusion coefficient measured in m^2^/s.

*Brain lactate*, which represents the ability to produce lactate, was measured using MR spectroscopy under normoxia and hypoxia [33,34]. The ability to respond to hypoxia with an increase in lactate in relation to group (TG or CG) allocation was assessed, with brain lactate measured in mmol/mL of brain tissue.

The magnitudes of the BOLD response and the CBF response (from pCASL) to *visual stimulation* in relation to group (TG or CG) allocation were assessed [35].

A *process evaluative approach* was adopted. Using a SWOT analytical framework [41,42], patients and researchers were invited to reflect on the perceived experiences of taking part in the different processes of the project. The approach conceptualized PPCSs as a complex phenomenon requiring participants’ real-time experiential input [24] to inform sense-making across all project processes and to generate recommendations for future projects. This evaluation was conducted at week 12.

The *experiences of patients with PPCSs* were assessed. A descriptive phenomenological approach [24] was used to describe patients’ specific experiences and perceptions of symptomatology and the physical capabilities prioritizing the patients’ own descriptions and nuances of living with PPCSs [43,44]. This exploration adhered to a humanistic orientation, using first-person interviews with a strategic selection of included patients. These interviews were also used to pave the way for the second round of interviews, to which all participants were invited. For this second round, a semistructured interview guide was developed based on the initial interviews and with the aim of generating data for grounded theory analysis [25,26].

Overviews of all outcomes and time points in the trial can be seen in Tables1  2.

### Adverse Events

Adverse events, adherence, and patient safety were monitored continuously throughout the trial. Withdrawal from the trial was considered if an adverse event occurred, adherence deteriorated substantially, or a serious illness arose such that the patient’s safety or the scientific integrity of their continued involvement could no longer be ensured. Decisions regarding withdrawal were made on a case-by-case basis, considering the nature, severity, and potential impact of the event on both patient safety and trial validity. All events relevant to these evaluations, including symptom exacerbations, missed sessions, safety-related concerns noted during supervised training sessions, and medical issues reported by the patient, were documented and revised. The project leader (BWL) was responsible for assessing each situation and determining the appropriate course of action, including involvement of medical doctors and withdrawal of the patient from the trial.

### Data Management

All data, including questionnaire data, were managed securely and in full compliance with the General Data Protection Regulation (GDPR) and the Danish Data Protection Agency’s guidelines for the protection of personal data. All data were collected electronically and entered directly during physical testing and training, as well as during MRI scans. Data collected through mail correspondence were entered directly by patients when they submitted responses via secure mail and were stored on secure drives at the trial sites. For qualitative interviews and process evaluations, data were recorded electronically and transcribed by project assistants, and the original recordings were subsequently deleted. All data were pseudonymized, and the decryption key was stored separately from the dataset. Access to the data was controlled and provided by Rigshospitalet and University College Copenhagen. All data were stored on secure drives at the trial sites. Written consent forms were stored in locked cabinets within a secured room, and the forms will be retained for 5 years following the completion of the RCT.

### Sample Size Calculation

Based on previous research [14], a clinically meaningful change in exercise tolerance on the BCBT has not been formally defined using a minimal clinically important difference. Therefore, a clinically meaningful difference was prespecified based on a combination of the empirical variability and functional interpretation of the test. In the original BCBT validation study, patients demonstrated a mean time to symptom provocation of 14.6 (SD 6.0) minutes, whereas healthy controls showed a mean time of 27.2 (SD 5.2) minutes, indicating a disease-related impairment of approximately 12‐13 minutes. A between-group difference of 6 minutes was therefore defined as clinically meaningful, corresponding to approximately half of this observed deficit, 1 SD of patient performance, and completion of 3 additional workload stages on the BCBT, which progresses in 2-minute increments.

Assuming a common SD of 6 minutes, a between-group difference of 6 minutes corresponds to a standardized effect size of 1.0. With a 2-sided type 1 error rate of 5% and a statistical power of 90%, at least 22 participants were required in each group.

Relevant uncertainty measurements for BBB permeability, spectroscopy, lactate, and diffusion were already available for subproject 3, for both paired and unpaired data. Specifically, for BBB permeability, we set the standard difference to 1 (effect size) based on our previous investigation of the reproducibility of BBB permeability [45], and setting the significance level to 1% and the power to 90%, 27 participants were required in each group. Considering a tentative dropout rate of 30%, we aimed to recruit a total of 70 participants in the study.

### Statistical Analysis

The primary outcome of this RCT study is an increase in time to symptom exacerbation during the BCBT from before to after the 12-week intervention. To evaluate this effect while avoiding sensitivity to false-positive error from repeated measurements on the same participant, we will use a linear mixed model. To assess the effect of the intervention, a regressor indicating either pre- or postintervention (Time) and a regressor indicating group allocation (Group) will be included as fixed effects. Participant identification will be included as a random effect (*u*) to account for repeated measurements. Sex and age may influence these results, and they will be included as fixed effects. The additional effect of group allocation and pre- and postintervention on the results of the BCBT will be included as an interaction term (Group_i_ and Time_ij_). This interaction term will provide the main result of the RCT study. The model is formulated as follows:


$$\begin{array}{c} Y_{ij}=\beta_{0}+\beta_{1}{\text{Group}}_{i}+\beta_{2}{\text{Time}}_{ij}+\beta_{3}({\text{Group}}_{i}\times {\text{Time}}_{ij})\\ +\beta_{4}{\text{Age}}_{i}+\beta_{5}{\text{Sex}}_{i}+u_{i} \end{array}$$

where *Y* is the duration (in minutes) of exercise on the bicycle until symptom provocation and *β*s are regression coefficients, with *β*_3_ representing the primary result of the RCT. The subscript *i* denotes the subject, and the subscript *j* denotes pre- or postintervention measurement.

For MRI parameters, 2 main tests will be performed for the baseline data. These analyses will be repeated after the 12-week intervention period and will include the intervention as a regressor, as explained in the following text. At baseline, the CMRO2 level (overall oxygen consumption of the brain) between healthy control participants and patients with PPCSs will be tested using ordinary least squares regression adjusted for age and sex, with heteroscedasticity-consistent SEs to account for possible nonconstant error variance. To investigate whether the physiological response to hypoxic exposure differs between patients with PPCSs and healthy controls, we will test whether the change in CMRO2 from hypoxic exposure differs between the groups. Initially, we will assess differences at baseline (preintervention) using a mixed linear regression as follows:


$$\begin{array}{c} Y_{ij}=\beta_{0}+\beta_{1}{\text{Group}}_{i}+\beta_{2}{\text{SaO}}_{2,ij}+\beta_{3}({\text{Group}}_{i}\times {\text{SaO}}_{2,ij})\\ +\beta_{4}{\text{Age}}_{i}+\beta_{5}{\text{Sex}}_{i}+u_{i} \end{array}$$

where *Y*_*ij*_ denotes CMRO2, Group refers to either healthy controls or patients with PPCSs, and SaO2 is the actual oxygen saturation level in blood measured during the experiment (ie, during normoxia and hypoxia). Thus, we take the actual individual level of blood desaturation into account. The regression value *β*_3_ of the interaction term provides an inference regarding the patient’s ability to maintain CMRO2 under hypoxic stress conditions. After the 12-week intervention period, only patients will be tested for a possible change in their ability to maintain CMRO2 during the hypoxic exposure. Patients will be split according to group allocation (CG or TG), and the model is formulated as follows:


$$\begin{array}{c} Y_{ijk}=\beta_{0}+\beta_{1}{\text{Group}}_{i}+\beta_{2}{\text{SaO}}_{2,ijk}+\beta_{3}{\text{Time}}_{jk}\\ +\beta_{4}({\text{Group}}_{i}\times {\text{SaO}}_{2,ijk}\times {\text{Time}}_{jk})+\beta_{5}{\text{Age}}_{i}+\beta_{6}{\text{Sex}}_{i}+u_{i} \end{array}$$

where Time_ijk_ codes for pre- or postintervention (*k*) and Group codes for either the CG or TG, with the other parameters being as described above. The interaction term infers information on whether time (pre- or postintervention) has any impact on the group-specific CMRO2 response. Comparison of BBB permeability between patients with PPCSs and healthy controls will be performed using a linear regression model adjusted for age and sex, with heteroscedasticity-consistent SEs to account for possible nonconstant error variance. For exploratory regional analysis of BBB permeability across multiple brain parcels, Benjamini-Hochberg false discovery rate correction will be applied to control for multiple comparisons.

Structural equation modeling (SEM) and latent class analysis (LCA) will be used to identify latent variables and subgroups associated with PPCSs. Both approaches will be applied in an exploratory framework to generate hypotheses for future research. In the SEM analyses, observed variables will be treated as manifestations of an underlying normally distributed latent construct representing PPCS severity. The model will include the summed Rivermead score, time cycled and maximal workload during the BCBT, MRI-based CMRO2 measurements under normoxia and hypoxia, and BBB permeability. The latent variable will be scaled to the summed Rivermead score to facilitate interpretation. By integrating information across multiple domains, we aim to obtain a more comprehensive and sensitive representation of symptom severity than can be obtained with any single measure alone. Conceptually or procedurally related indicators (eg, fatigue-related items, BCBT variables, and MRI measures) will be modeled to account for residual correlations by including terms describing local dependence. This is performed to avoid inflating latent factor loadings or identifying spurious subgroups driven by unmodeled shared variance. In the LCA analyses, observed variables will be treated as indicators of an underlying categorical latent variable. Patients will thus be grouped according to their multivariate response profiles. The number of classes will be estimated using information criteria (Akaike information criterion and Bayesian information criterion) and by excluding solutions with very small or clinically uninterpretable classes. Given the limited sample size, we will restrict models to a maximum of four classes. For each class, we will estimate the expected response pattern and assess whether the resulting classes have meaningful clinical interpretations. Associations between latent outcomes and external covariates, such as age and sex, will be examined post hoc, with covariates treated as external variables rather than incorporated directly into the latent variable models. This includes assessing whether patients are exercise-intolerant, defined by both clinical criteria and self-reported experience. Finally, we will extend the models to incorporate both baseline and follow-up data. In the SEM analyses, we will estimate the effect of the intervention on the latent PPCS construct, while in the LCA analyses, we will test whether the intervention effect differs across latent classes.

Statistical modeling will be handled externally at the Institute for Biostatistics, University of Copenhagen. In this large study, we have included other a priori defined measures of distinct types, and therefore, further testing will be performed. We treat these tests as hypothesis-generating for further studies and consider that corrections for multiple testing are irrelevant, as discussed previously [46,47].

### Missing Data

For the primary outcome, an intention-to-treat analysis including all patients with at least baseline test data as a repeated observation of the dependent variable will be performed using a linear mixed model. Missing data will be assumed to be missing at random, and there will be no imputations [48]. A complementary per-protocol analysis will also be performed, with the inclusion criteria reported in detail. For the primary outcome analysis, participants will be included regardless of the availability of secondary outcomes, including MRI data.

### Ethical Considerations

This study has been conducted in accordance with the principles of the Declaration of Helsinki, and the protocol was approved by the Research Ethics Committee in the Capital Region of Denmark (reg. H-21047857; January 2022) and cannot be amended without reapplication. Furthermore, this study has been conducted in adherence to the study protocol and International Council for Harmonisation Good Clinical Practice guidelines, following applicable Danish and European law. The study does not fall under the scope of external monitoring, as it does not involve medical products or medical devices. Patients were covered by insurance through Rigshospitalet and the Danish Patient Compensation Association. Participation was voluntary, and inclusion required informed consent that could be withdrawn at any time by the participant.

### Protocol Amendments

The ethics protocol was extended for 1 year to accommodate the inclusion of 2-3 patients per week until December 2025.

## Results

**Figure 1.**
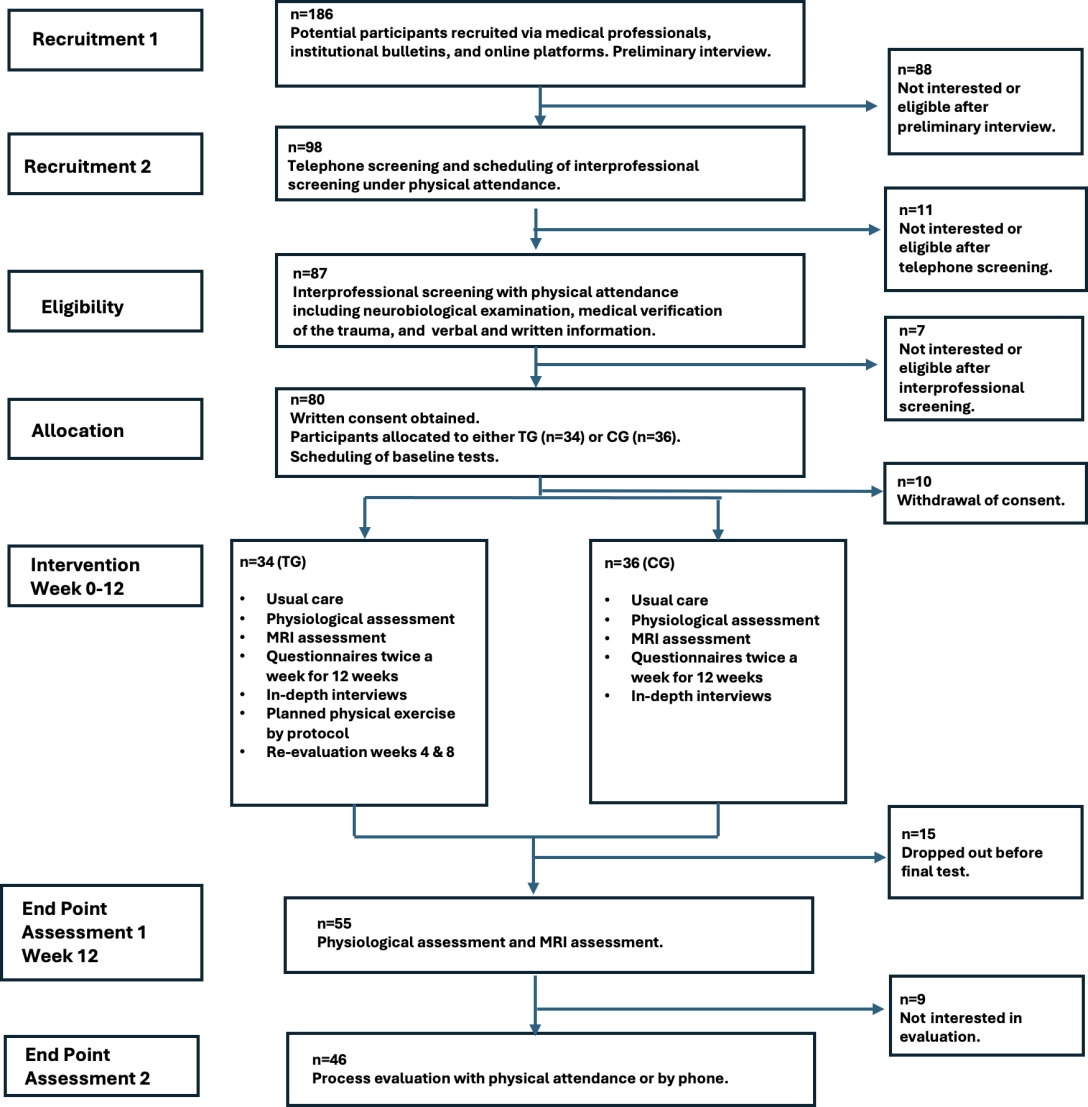
Study flowchart providing an overview of the study and patient recruitment and allocation. CG: control group; MRI: magnetic resonance imaging; TG: treatment group.

The study was funded in December 2022. The ethically approved study protocol (H-21047857) was registered on ClinicalTrials.gov (NCT05785000), with study initiation on February 1, 2023. Enrollment and randomization of 70 patients were completed in March 2025. An overview of the study and patient recruitment and allocation is shown in Figure 1. The 12-week postintervention assessments concluded in August 2025, and data analysis is currently being performed, with finalization expected in 2026. The results will be reported in separate manuscripts in 2027.

Manuscripts reporting the study outcomes will be prepared for submission to peer-reviewed scientific journals, adhering to authorship guidelines. The following seven publications are planned (tentative titles):

1. A 12-week exercise intervention improves physical capacity in patients with prolonged post-concussion symptoms: an RCT study – the REPCon project
2. Reduced cerebral oxygen metabolism in persistent post-concussion syndrome after mild traumatic brain injury
3. The symptom complexity and intolerance in persistent post-concussion symptoms: insights from a controlled clinical study
4. Persistent post-concussion symptoms and clinical trial participation: a constructivist grounded theory study
5. Blood-brain barrier permeability is (not) increased in patients with prolonged post- concussion symptoms
6. A 12-week exercise intervention does (not) improve reduced brain oxygen metabolism in patients with prolonged post-concussion syndrome: an RCT study – the REPCon project
7. Understanding the PPCS phenomenon through combined data using structural equation modeling and latent group statistics

Trial results will be presented at conferences and meetings and will be made accessible to the public through appropriate and relevant media, including negative, positive, or inconclusive results. In addition, findings will be communicated through presentations and dialogue with clinical practice stakeholders and patient organizations to ensure transparency, foster engagement, and support the translation of results into real-world settings.

## Discussion

We have presented the protocol for an RCT that evaluates the effect of an individualized physical aerobic graded exercise intervention in adults with PPCSs. The study has also used an intensive battery of physical and MRI-based examinations, as well as personal interviews and process evaluations. To our knowledge, this combined research methodology is scarce and could lead to a novel and integrated understanding of the complex phenomenon of persistent symptomatology and functional disabilities following PPCSs, where traumatology involves substantial neurological impairment. However, no single generally accepted pathophysiological component has been identified for this illness; rather, patients experience physical, neurological, cognitive, and perceptual inabilities. Combining an intervention study with an MRI-based investigation of brain physiology provides an opportunity to disclose the interaction between physical activity and brain function. While there is evidence for a beneficial effect of subsymptomatic exercise rehabilitation in patients with PPCSs [17,18], the impact on brain function is less known. Several MRI studies have pointed to microstructural abnormalities and possible changes in the brain functional connectivity network, using diffusion tensor MRI [49,50] and BOLD MRI [51,52]. Furthermore, autonomic dysfunction due to disturbances in brainstem function is of particular interest [11]. Clausen et al [53] and Mutch et al [54] have mentioned reduced carbon dioxide sensitivity and a lack of ability to increase ventilation during physical activity in patients with PPCSs. Reduced sensitivity to carbon dioxide results in abnormally high CBF during physical activity and may explain the exercise intolerance seen in many patients with PPCSs [53]. The findings by Clausen et al [53] underscore the importance of investigating brain physiology in this population. In this study, we have chosen a brain “stress” test consisting of a hypoxic challenge. This setup provides details regarding brain oxygen consumption, cerebrovascular reactivity, and the brain’s ability to increase glycolysis, which can manifest as an increase in brain lactate during hypoxia. The responses in young healthy individuals include maintained oxygen consumption, a pronounced increase in brain blood flow, and lactate production [30,33]. In older people, the ability to increase brain blood flow is diminished, and this results in a small but significant decrease in oxygen consumption [32]. Patients with sleep apnea show a reduced CBF response to hypoxia, and continuous positive airway pressure treatment has been found to normalize this pattern [29]. Patients with type 1 diabetes show a normal CBF response to hypoxia but significantly reduced lactate production during hypoxia, indicating impaired glycolytic activity [55]. In patients with multiple sclerosis (MS), the expected increase in blood flow is reduced, and a pronounced fall in oxygen consumption occurs (Vestergaard MB, MSc, unpublished data, May 2026). In this study on MS, vascular reactivity to carbon dioxide was tested and found to be normal. Hypercapnia and hypoxia induce cerebral vasodilation through different mechanisms [56]. In hypercapnia, carbon dioxide lowers local pH, which opens K^+^ channels in vascular smooth muscle cells, reduces Ca²^+^ influx, and thereby promotes vasodilation. Hypoxia induces vasodilation through a complex process that likely involves multiple mechanisms. Hypoxia stimulates nitric oxide release from nitric oxide synthase in both endothelium cells and neurons. Nitric oxide activates several enzymatic pathways, ultimately resulting in vasodilation and CBF increase. Thus, the hypoxic brain “stress” test investigates fundamental elements of brain physiology. The cerebrovascular response to hypoxia has not been systematically investigated in patients with PPCSs. The motivation to study BBB integrity is based on previous findings of increased BBB leakiness in both experimental mTBI animal models and patients with mTBI and PPCSs [15,57-60]. The BBB is important for maintaining proper brain function and a precise biochemical brain tissue environment while serving as a barrier against the entrance of toxic molecules, bacteria, and viruses. The function of the BBB is also highly related to vascular reactivity. In this study, we have used DCE MRI, which is a highly sensitive method that has been used previously for the detection of subtle leakiness in patients with MS [61-63].

This study is unique because it allows us to evaluate how an intervention consisting of subsymptomatic exercise can modify the anticipated disturbed brain physiology in patients with PPCSs. Moreover, the study aims to integrate novel neuropathophysiological insights with patient perspectives on the short- and long-term impacts of PPCSs on physical, neurobiological, and social functioning.

The study has some limitations. First, patients were not blinded to group allocation, and a desire to switch to the other group could have introduced unwanted bias, leading to lower compliance and poor protocol adherence. Second, patients were required to complete 4 MRI sessions (up to 6 hours) in a noisy environment, and for some patients with PPCSs, this might be challenging and provoke an increased symptom burden. Thus, it was decided to modify the approach and consider not completing all 4 MRI sessions as acceptable. Third, patients in the TG were required to meet in person twice weekly for the 12-week aerobic exercise protocol, and this could potentially affect the primary outcome. To support participation compliance, flexible scheduling was offered. Furthermore, practical factors, such as traffic delays, canceled trains, and seasonal illnesses, might have made it difficult for patients to attend the planned sessions. Finally, participants in the TG received a higher level of supervision and clinical contact compared to those in the CG, which might have introduced a risk of attention bias.

This study is innovative in its approach to patients with PPCSs, focusing on the effect of an individualized exercise regimen and exploring the neurofunctional underpinnings of symptom improvements and the patient perceptions of dealing with PPCSs. The findings are anticipated to contribute greatly to the field of PPCS management, potentially transforming current rehabilitation practices, alleviating symptom burden, and improving recovery from mTBI.

## Supplementary material

10.2196/93795 Checklist 1 SPIRIT checklist.

## Abbreviations

BBB: blood-brain barrier
BCBT: Buffalo Concussion Bike Test
BOLD: blood oxygenation level–dependent
CBF: cerebral blood flow
CG: control group
CMRO2: cerebral metabolic rate of oxygen
DCE: dynamic contrast-enhanced
GDPR: General Data Protection Regulation
HR: heart rate
HRt: heart rate at symptom threshold
LCA: latent class analysis
MR: magnetic resonance
MRI: magnetic resonance imaging
MRS: magnetic resonance spectroscopy
MS: multiple sclerosis
mTBI: mild traumatic brain injury
pCASL: pseudo-continuous arterial spin labeling
PPCS: persistent postconcussion symptom
RCT: randomized controlled trial
REPCon: Rehabilitation in Postconcussion Symptoms
RPE: Rating of Perceived Exertion
rpm: revolutions per minute
RPQ: Rivermead Post-Concussion Symptoms Questionnaire
SaO2: arterial saturation
SEM: structural equation modeling
SPIRIT: Standard Protocol Items: Recommendations for Interventional Trials
SWOT: strengths, weaknesses, opportunities, and threats
TBI: traumatic brain injury
TG: training group
VAS: Visual Analog Scale
